# Dr. Oliver Wendell Holmes on Homœpathy and Its Kindred Delusions

**Published:** 1842-04-23

**Authors:** 


### BIBLIOGRAPHICAL NOTICES.

*Homœopathy, and its kindred Delusions; two Lectures delivered before the Boston Society for the Diffusion of Useful Knowledge.* By OLIVER WENDELL HOLMES, M. D. Boston, 1842.

We commend Dr. Holmes' book, particularly, to the few physicians of honest minds, who have been led away by the *ignis fatuus* of some isolated facts, to a sort of half-adoption of the principles of homœopathy. It is a most candid examination as well as thorough refutation of the new doctrine, and of the array of facts by which it is supported. As a proper preamble to the discussion of the prevailing delusion of the day, Dr. Holmes touches upon some of the popular fallacies of former days—now almost forgotten, but in their time received by some of the wisest and most distinguished of mankind. Of these, he notices especially, the Royal cure of the King's evil, the Unguentum Armentarium or Weapon ointment, Bishop Berkeley's Tar-water, and the Metallic Tractors, or Perkinism. The three former are of somewhat antiquated date, but the latter is of more recent notoriety. Perkinism, which flourished about forty years ago, consisted in the cure of diseases, by the local application of *metallic tractors*—two pieces of metal, one apparently iron and the other brass, about three inches long, blunt at one end and pointed at the other. They were applied by drawing them lightly over the part affected for about twenty minutes. The metallic tractors were the inventions of Dr. Elisha Perkins, of Norwich, Connecticut, who in 1796, took out a patent for them. They soon came into vogue, not only in this country, but in Europe, and for a while, enjoyed the patronage of a great number of distinguished individuals. Perkinism, like all quackeries, in time died a

*Of salep, gum tragacanth, sago, each four ounces, of cochineal half a drachm, and of prepared chalk, an ounce.

natural death. Dr. Holmes has disinterred it, to illustrate the more modern delusion, homœopathy.

Homœopathy he has of course very thoroughly used up—demonstrating the absurdity of its doctrines, and making clear the falseness of its facts. He discusses the subject with calmness, avoiding ridicule, and confining himself to argumentative refutation. This is certainly the right mode of handling such subjects, at least the only one likely to do good. We are satisfied that Dr. Holmes' lectures have done and will do much good.

